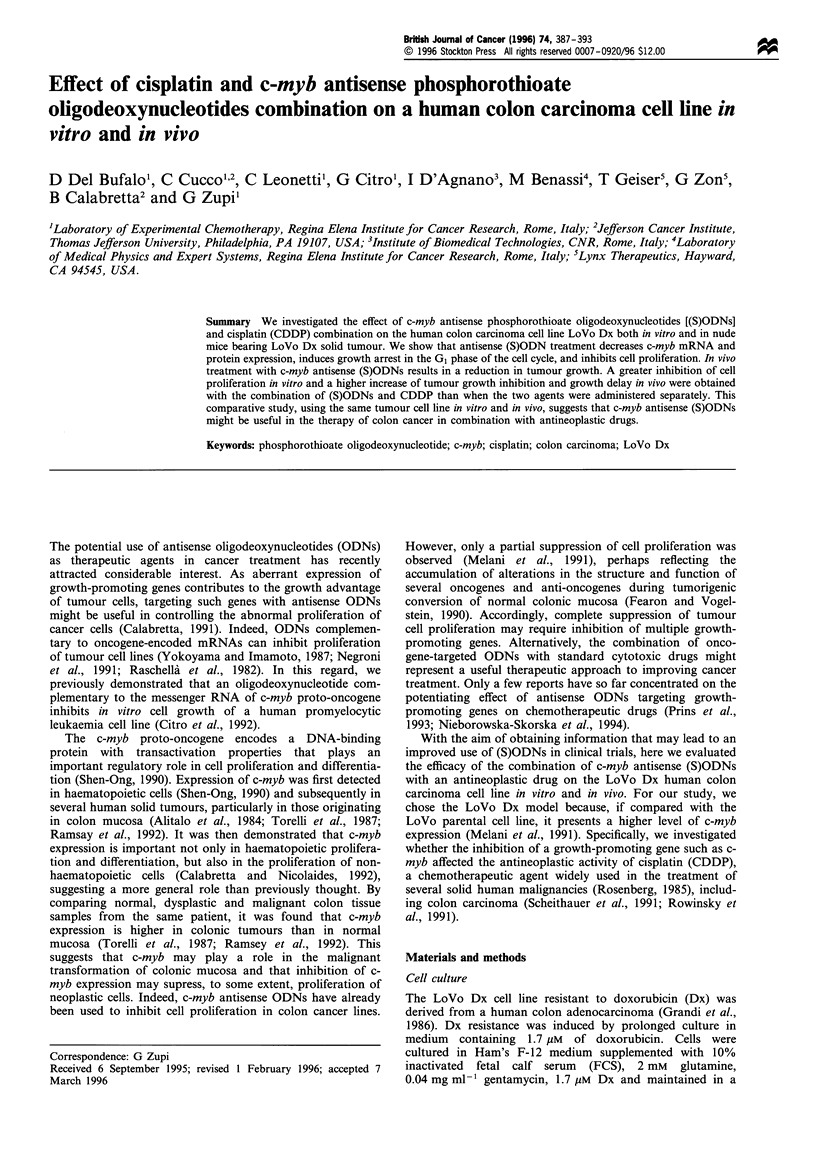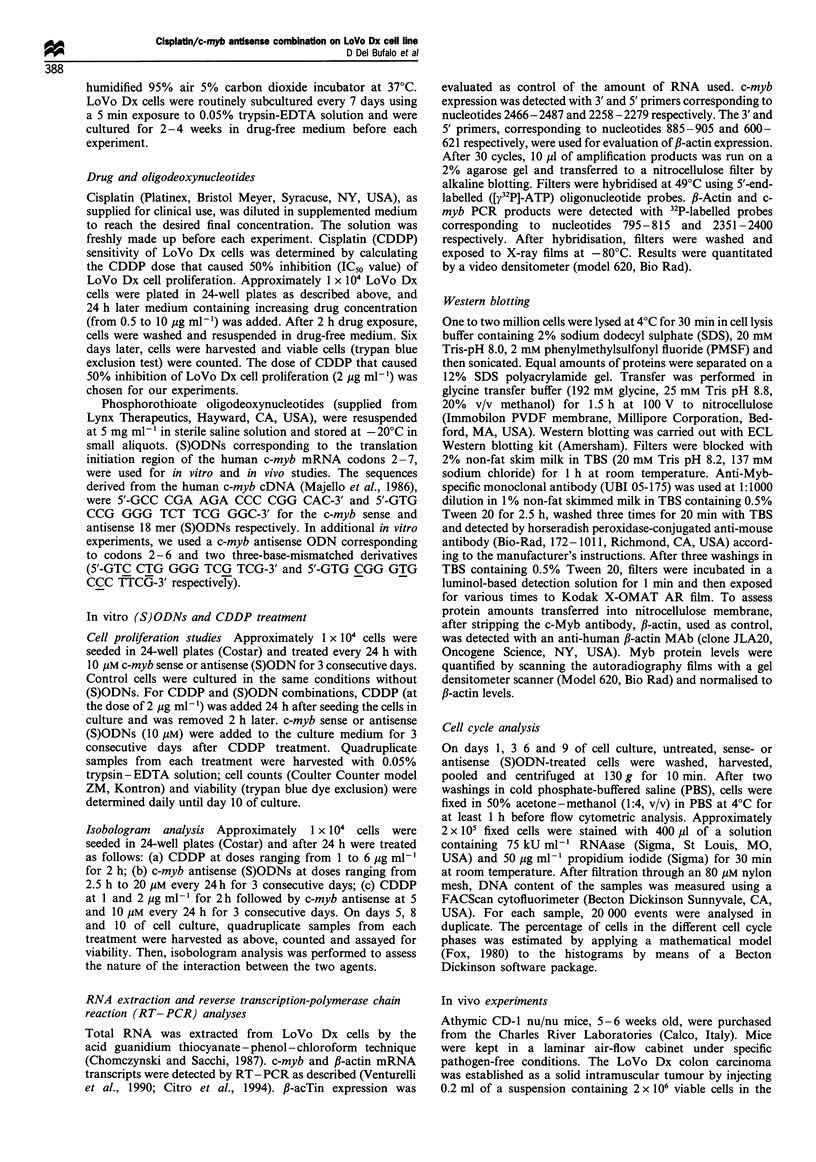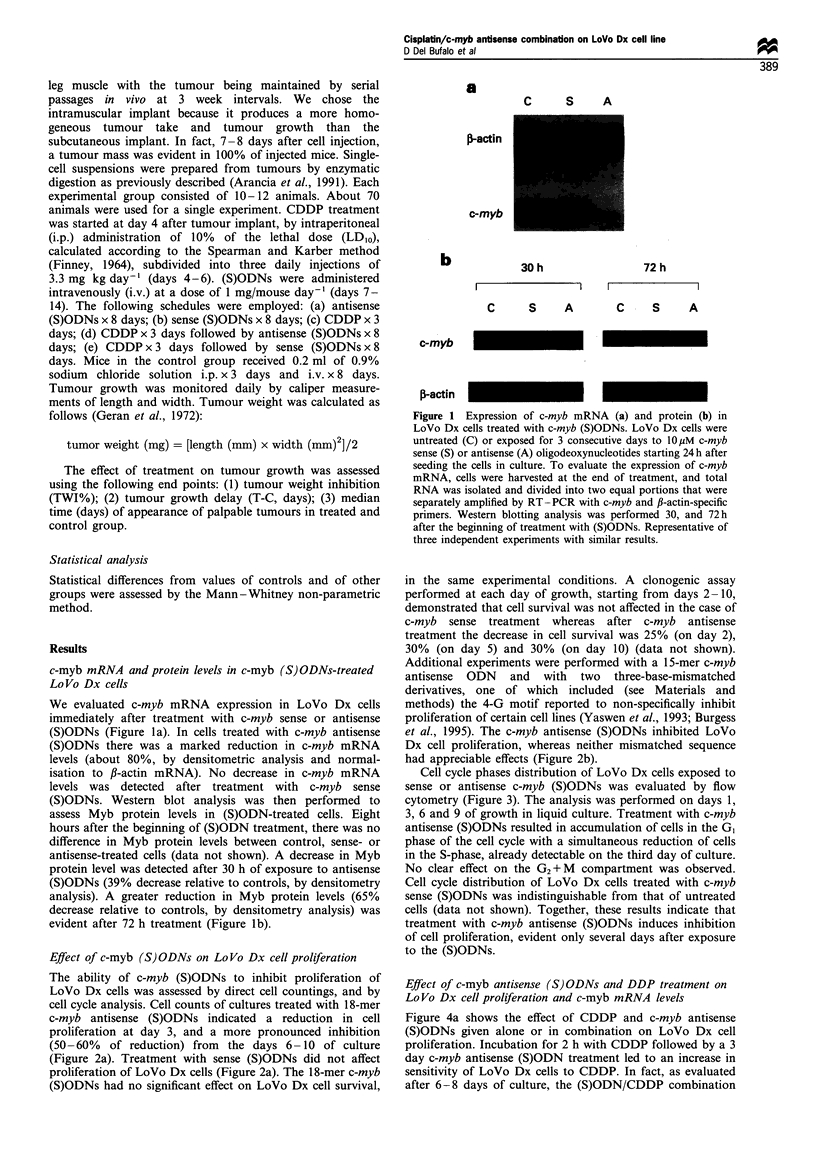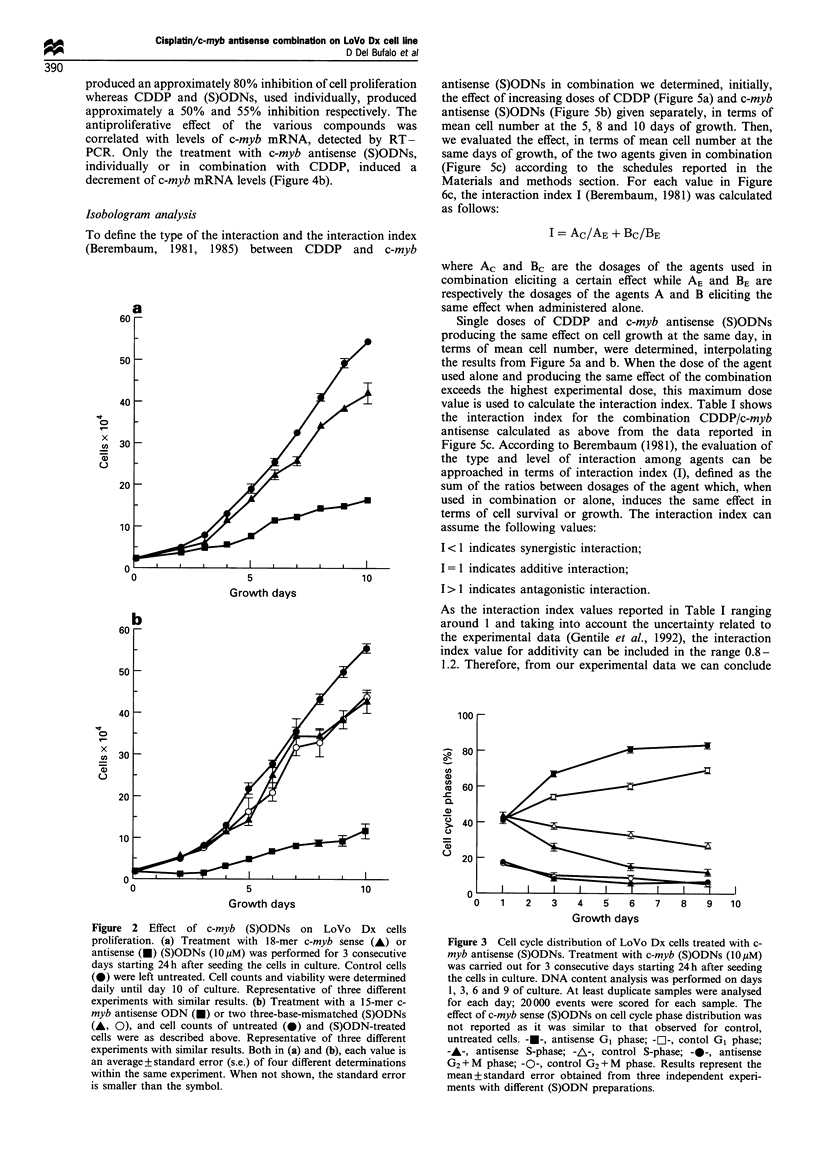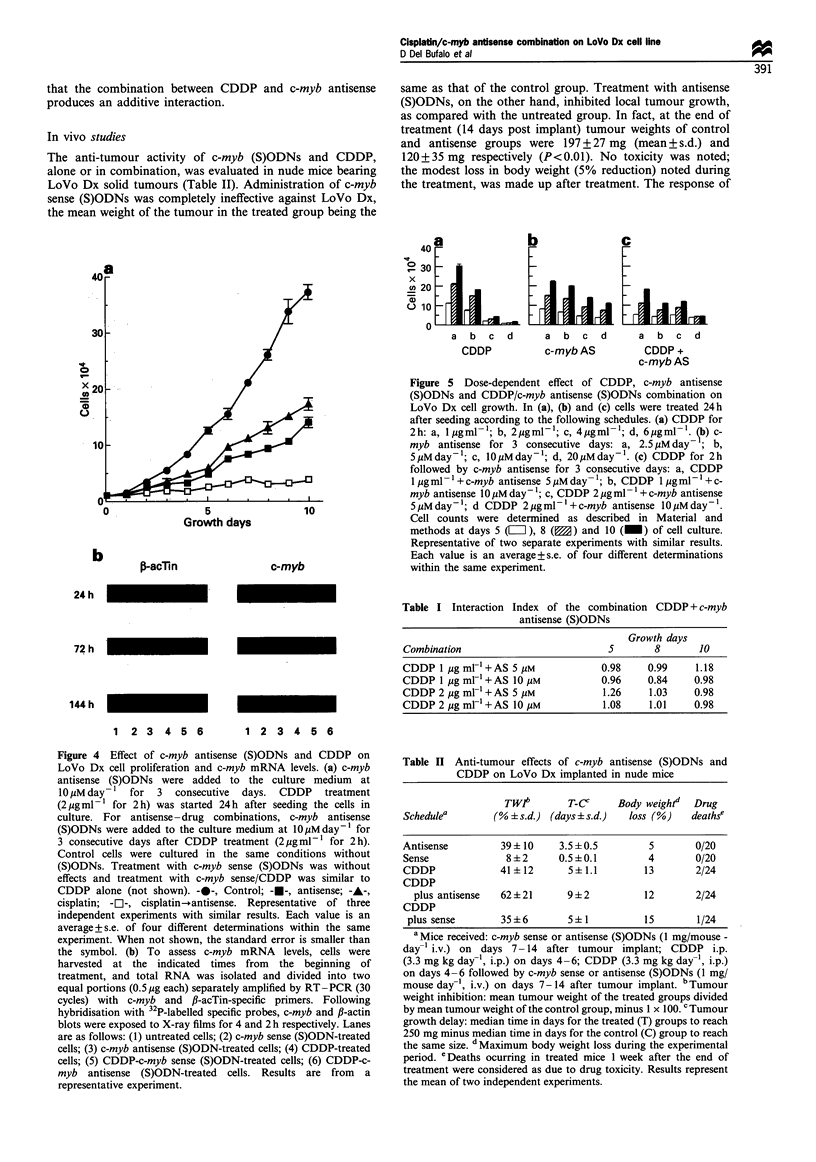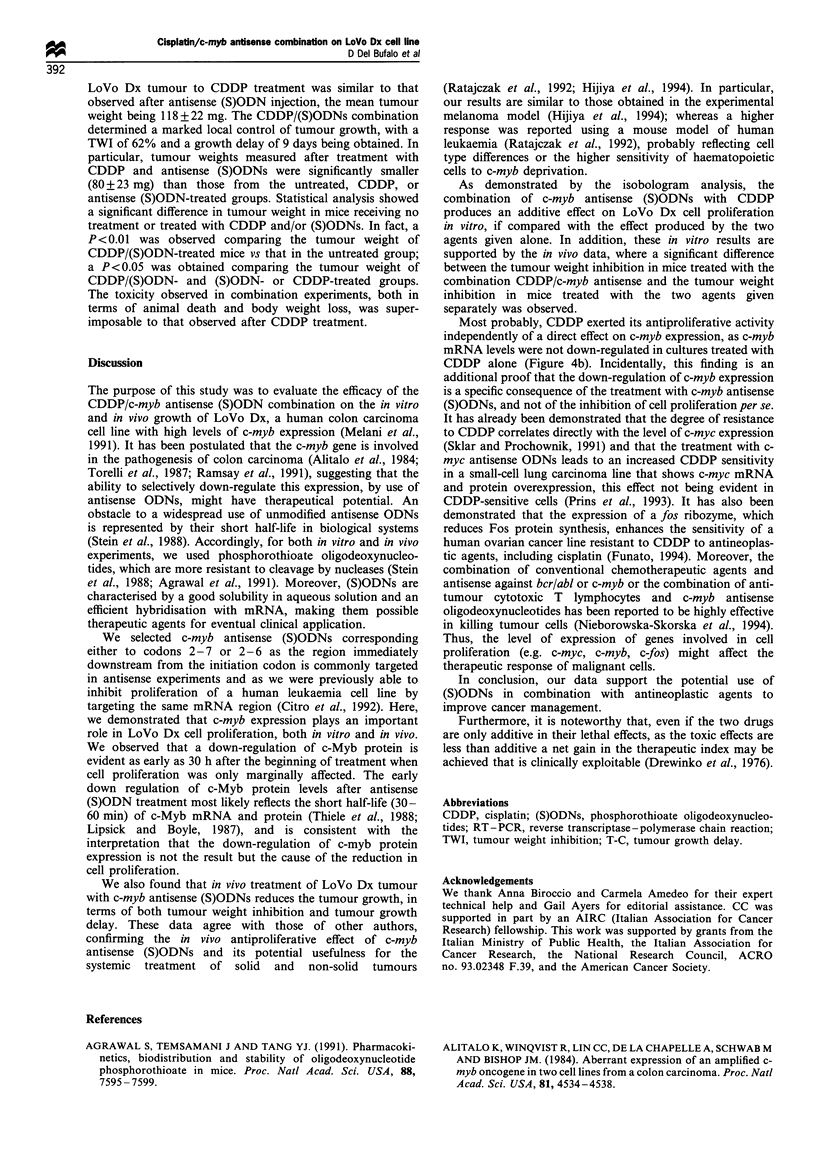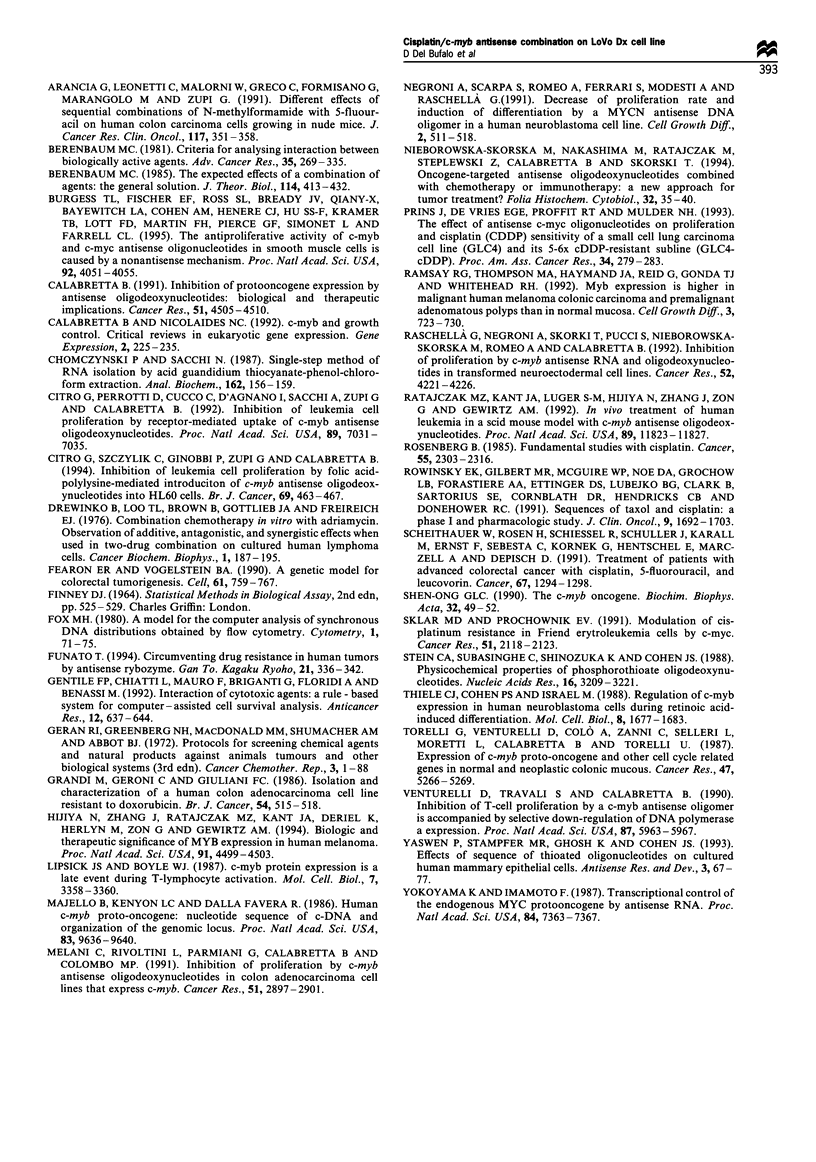# Effect of cisplatin and c-myb antisense phosphorothioate oligodeoxynucleotides combination on a human colon carcinoma cell line in vitro and in vivo.

**DOI:** 10.1038/bjc.1996.370

**Published:** 1996-08

**Authors:** D. Del Bufalo, C. Cucco, C. Leonetti, G. Citro, I. D'Agnano, M. Benassi, T. Geiser, G. Zon, B. Calabretta, G. Zupi

**Affiliations:** Laboratory of Experimental Chemotherapy, Regina Elena Institute for Cancer Research, Rome, Italy.

## Abstract

**Images:**


British Journal of Cancer (1996) 74, 387-393
© 1996 Stockton Press All rights reserved 0007-0920/96 $12.00

### Effect of cisplatin and c-*myb* antisense phosphorothioate oligodeoxynucleotides combination on a human colon carcinoma cell line *in vitro* and *in vivo*

D Del Bufalo[1], C Cucco[1,2], C Leonetti[1], G Citro[1], I D'Agnano[3], M Benassi[4], T Geiser[5], G Zon[5], B Calabretta[2] and G Zupi[1]

*[1]Laboratory of Experimental Chemotherapy, Regina Elena Institute for Cancer Research, Rome, Italy; [2]Jefferson Cancer Institute, Thomas Jefferson University, Philadelphia, PA 19107, USA; [3]Institute of Biomedical Technologies, CNR, Rome, Italy; [4]Laboratory of Medical Physics and Expert Systems, Regina Elena Institute for Cancer Research, Rome, Italy; [5]Lynx Therapeutics, Hayward, CA 94545, USA.*

**Summary** We investigated the effect of c-*myb* antisense phosphorothioate oligodeoxynucleotides [(S)ODNs] and cisplatin (CDDP) combination on the human colon carcinoma cell line LoVo Dx both *in vitro* and in nude mice bearing LoVo Dx solid tumour. We show that antisense (S)ODN treatment decreases c-*myb* mRNA and protein expression, induces growth arrest in the $G_1$ phase of the cell cycle, and inhibits cell proliferation. *In vivo* treatment with c-*myb* antisense (S)ODNs results in a reduction in tumour growth. A greater inhibition of cell proliferation *in vitro* and a higher increase of tumour growth inhibition and growth delay *in vivo* were obtained with the combination of (S)ODNs and CDDP than when the two agents were administered separately. This comparative study, using the same tumour cell line *in vitro* and *in vivo*, suggests that c-*myb* antisense (S)ODNs might be useful in the therapy of colon cancer in combination with antineoplastic drugs.

**Keywords:** phosphorothioate oligodeoxynucleotide; c-*myb*; cisplatin; colon carcinoma; LoVo Dx

The potential use of antisense oligodeoxynucleotides (ODNs) as therapeutic agents in cancer treatment has recently attracted considerable interest. As aberrant expression of growth-promoting genes contributes to the growth advantage of tumour cells, targeting such genes with antisense ODNs might be useful in controlling the abnormal proliferation of cancer cells (Calabretta, 1991). Indeed, ODNs complementary to oncogene-encoded mRNAs can inhibit proliferation of tumour cell lines (Yokoyama and Imamoto, 1987; Negroni *et al.*, 1991; Raschellà *et al.*, 1982). In this regard, we previously demonstrated that an oligodeoxynucleotide complementary to the messenger RNA of c-*myb* proto-oncogene inhibits *in vitro* cell growth of a human promyelocytic leukaemia cell line (Citro *et al.*, 1992).

The c-*myb* proto-oncogene encodes a DNA-binding protein with transactivation properties that plays an important regulatory role in cell proliferation and differentiation (Shen-Ong, 1990). Expression of c-*myb* was first detected in haematopoietic cells (Shen-Ong, 1990) and subsequently in several human solid tumours, particularly in those originating in colon mucosa (Alitalo *et al.*, 1984; Torelli *et al.*, 1987; Ramsay *et al.*, 1992). It was then demonstrated that c-*myb* expression is important not only in haematopoietic proliferation and differentiation, but also in the proliferation of non-haematopoietic cells (Calabretta and Nicolaides, 1992), suggesting a more general role than previously thought. By comparing normal, dysplastic and malignant colon tissue samples from the same patient, it was found that c-*myb* expression is higher in colonic tumours than in normal mucosa (Torelli *et al.*, 1987; Ramsey *et al.*, 1992). This suggests that c-*myb* may play a role in the malignant transformation of colonic mucosa and that inhibition of c-*myb* expression may supress, to some extent, proliferation of neoplastic cells. Indeed, c-*myb* antisense ODNs have already been used to inhibit cell proliferation in colon cancer lines. However, only a partial suppression of cell proliferation was observed (Melani *et al.*, 1991), perhaps reflecting the accumulation of alterations in the structure and function of several oncogenes and anti-oncogenes during tumorigenic conversion of normal colonic mucosa (Fearon and Vogelstein, 1990). Accordingly, complete suppression of tumour cell proliferation may require inhibition of multiple growth-promoting genes. Alternatively, the combination of oncogene-targeted ODNs with standard cytotoxic drugs might represent a useful therapeutic approach to improving cancer treatment. Only a few reports have so far concentrated on the potentiating effect of antisense ODNs targeting growth-promoting genes on chemotherapeutic drugs (Prins *et al.*, 1993; Nieborowska-Skorska *et al.*, 1994).

With the aim of obtaining information that may lead to an improved use of (S)ODNs in clinical trials, here we evaluated the efficacy of the combination of c-*myb* antisense (S)ODNs with an antineoplastic drug on the LoVo Dx human colon carcinoma cell line *in vitro* and *in vivo*. For our study, we chose the LoVo Dx model because, if compared with the LoVo parental cell line, it presents a higher level of c-*myb* expression (Melani *et al.*, 1991). Specifically, we investigated whether the inhibition of a growth-promoting gene such as c-*myb* affected the antineoplastic activity of cisplatin (CDDP), a chemotherapeutic agent widely used in the treatment of several solid human malignancies (Rosenberg, 1985), including colon carcinoma (Scheithauer *et al.*, 1991; Rowinsky *et al.*, 1991).

### Materials and methods

### *Cell culture*

The LoVo Dx cell line resistant to doxorubicin (Dx) was derived from a human colon adenocarcinoma (Grandi *et al.*, 1986). Dx resistance was induced by prolonged culture in medium containing 1.7 $\mu$M of doxorubicin. Cells were cultured in Ham's F-12 medium supplemented with 10% inactivated fetal calf serum (FCS), 2 mM glutamine, 0.04 mg $ml^{-1}$ gentamycin, 1.7 $\mu$M Dx and maintained in a

Correspondence: G Zupi
Received 6 September 1995; revised 1 February 1996; accepted 7 March 1996

humidified 95% air 5% carbon dioxide incubator at 37°C. LoVo Dx cells were routinely subcultured every 7 days using a 5 min exposure to 0.05% trypsin-EDTA solution and were cultured for 2–4 weeks in drug-free medium before each experiment.

*Drug and oligodeoxynucleotides*

Cisplatin (Platinex, Bristol Meyer, Syracuse, NY, USA), as supplied for clinical use, was diluted in supplemented medium to reach the desired final concentration. The solution was freshly made up before each experiment. Cisplatin (CDDP) sensitivity of LoVo Dx cells was determined by calculating the CDDP dose that caused 50% inhibition ($IC_{50}$ value) of LoVo Dx cell proliferation. Approximately $1 \times 10^4$ LoVo Dx cells were plated in 24-well plates as described above, and 24 h later medium containing increasing drug concentration (from 0.5 to 10 $\mu g\ ml^{-1}$) was added. After 2 h drug exposure, cells were washed and resuspended in drug-free medium. Six days later, cells were harvested and viable cells (trypan blue exclusion test) were counted. The dose of CDDP that caused 50% inhibition of LoVo Dx cell proliferation (2 $\mu g\ ml^{-1}$) was chosen for our experiments.

Phosphorothioate oligodeoxynucleotides (supplied from Lynx Therapeutics, Hayward, CA, USA), were resuspended at 5 mg $ml^{-1}$ in sterile saline solution and stored at −20°C in small aliquots. (S)ODNs corresponding to the translation initiation region of the human c-*myb* mRNA codons 2–7, were used for *in vitro* and *in vivo* studies. The sequences derived from the human c-*myb* cDNA (Majello *et al*., 1986), were 5′-GCC CGA AGA CCC CGG CAC-3′ and 5′-GTG CCG GGG TCT TCG GGC-3′ for the c-*myb* sense and antisense 18 mer (S)ODNs respectively. In additional *in vitro* experiments, we used a c-*myb* antisense ODN corresponding to codons 2–6 and two three-base-mismatched derivatives (5′-GTC CTG GGG TCG TCG-3′ and 5′-GTG CGG GTG CCC TTCG-3′ respectively).

In vitro *(S)ODNs and CDDP treatment*

*Cell proliferation studies* Approximately $1 \times 10^4$ cells were seeded in 24-well plates (Costar) and treated every 24 h with 10 $\mu$M c-*myb* sense or antisense (S)ODN for 3 consecutive days. Control cells were cultured in the same conditions without (S)ODNs. For CDDP and (S)ODN combinations, CDDP (at the dose of 2 $\mu g\ ml^{-1}$) was added 24 h after seeding the cells in culture and was removed 2 h later. c-*myb* sense or antisense (S)ODNs (10 $\mu$M) were added to the culture medium for 3 consecutive days after CDDP treatment. Quadruplicate samples from each treatment were harvested with 0.05% trypsin–EDTA solution; cell counts (Coulter Counter model ZM, Kontron) and viability (trypan blue dye exclusion) were determined daily until day 10 of culture.

*Isobologram analysis* Approximately $1 \times 10^4$ cells were seeded in 24-well plates (Costar) and after 24 h were treated as follows: (a) CDDP at doses ranging from 1 to 6 $\mu g\ ml^{-1}$ for 2 h; (b) c-*myb* antisense (S)ODNs at doses ranging from 2.5 h to 20 $\mu$M every 24 h for 3 consecutive days; (c) CDDP at 1 and 2 $\mu g\ ml^{-1}$ for 2 h followed by c-*myb* antisense at 5 and 10 $\mu$M every 24 h for 3 consecutive days. On days 5, 8 and 10 of cell culture, quadruplicate samples from each treatment were harvested as above, counted and assayed for viability. Then, isobologram analysis was performed to assess the nature of the interaction between the two agents.

*RNA extraction and reverse transcription-polymerase chain reaction (RT–PCR) analyses*

Total RNA was extracted from LoVo Dx cells by the acid guanidium thiocyanate–phenol–chloroform technique (Chomczynski and Sacchi, 1987). c-*myb* and $\beta$-actin mRNA transcripts were detected by RT–PCR as described (Venturelli *et al*., 1990; Citro *et al*., 1994). $\beta$-acTin expression was evaluated as control of the amount of RNA used. c-*myb* expression was detected with 3′ and 5′ primers corresponding to nucleotides 2466–2487 and 2258–2279 respectively. The 3′ and 5′ primers, corresponding to nucleotides 885–905 and 600–621 respectively, were used for evaluation of $\beta$-actin expression. After 30 cycles, 10 $\mu$l of amplification products was run on a 2% agarose gel and transferred to a nitrocellulose filter by alkaline blotting. Filters were hybridised at 49°C using 5′-end-labelled ([$\gamma^{32}$P]-ATP) oligonucleotide probes. $\beta$-Actin and c-*myb* PCR products were detected with $^{32}$P-labelled probes corresponding to nucleotides 795–815 and 2351–2400 respectively. After hybridisation, filters were washed and exposed to X-ray films at −80°C. Results were quantitated by a video densitometer (model 620, Bio Rad).

*Western blotting*

One to two million cells were lysed at 4°C for 30 min in cell lysis buffer containing 2% sodium dodecyl sulphate (SDS), 20 mM Tris-pH 8.0, 2 mM phenylmethylsulfonyl fluoride (PMSF) and then sonicated. Equal amounts of proteins were separated on a 12% SDS polyacrylamide gel. Transfer was performed in glycine transfer buffer (192 mM glycine, 25 mM Tris pH 8.8, 20% v/v methanol) for 1.5 h at 100 V to nitrocellulose (Immobilon PVDF membrane, Millipore Corporation, Bedford, MA, USA). Western blotting was carried out with ECL Western blotting kit (Amersham). Filters were blocked with 2% non-fat skim milk in TBS (20 mM Tris pH 8.2, 137 mM sodium chloride) for 1 h at room temperature. Anti-Myb-specific monoclonal antibody (UBI 05-175) was used at 1:1000 dilution in 1% non-fat skimmed milk in TBS containing 0.5% Tween 20 for 2.5 h, washed three times for 20 min with TBS and detected by horseradish peroxidase-conjugated anti-mouse antibody (Bio-Rad, 172–1011, Richmond, CA, USA) according to the manufacturer's instructions. After three washings in TBS containing 0.5% Tween 20, filters were incubated in a luminol-based detection solution for 1 min and then exposed for various times to Kodak X-OMAT AR film. To assess protein amounts transferred into nitrocellulose membrane, after stripping the c-Myb antibody, $\beta$-actin, used as control, was detected with an anti-human $\beta$-actin MAb (clone JLA20, Oncogene Science, NY, USA). Myb protein levels were quantified by scanning the autoradiography films with a gel densitometer scanner (Model 620, Bio Rad) and normalised to $\beta$-actin levels.

*Cell cycle analysis*

On days 1, 3 6 and 9 of cell culture, untreated, sense- or antisense (S)ODN-treated cells were washed, harvested, pooled and centrifuged at 130 *g* for 10 min. After two washings in cold phosphate-buffered saline (PBS), cells were fixed in 50% acetone–methanol (1:4, v/v) in PBS at 4°C for at least 1 h before flow cytometric analysis. Approximately $2 \times 10^5$ fixed cells were stained with 400 $\mu$l of a solution containing 75 kU $ml^{-1}$ RNAase (Sigma, St Louis, MO, USA) and 50 $\mu g\ ml^{-1}$ propidium iodide (Sigma) for 30 min at room temperature. After filtration through an 80 $\mu$M nylon mesh, DNA content of the samples was measured using a FACScan cytofluorimeter (Becton Dickinson Sunnyvale, CA, USA). For each sample, 20 000 events were analysed in duplicate. The percentage of cells in the different cell cycle phases was estimated by applying a mathematical model (Fox, 1980) to the histograms by means of a Becton Dickinson software package.

In vivo *experiments*

Athymic CD-1 nu/nu mice, 5–6 weeks old, were purchased from the Charles River Laboratories (Calco, Italy). Mice were kept in a laminar air-flow cabinet under specific pathogen-free conditions. The LoVo Dx colon carcinoma was established as a solid intramuscular tumour by injecting 0.2 ml of a suspension containing $2 \times 10^6$ viable cells in the leg muscle with the tumour being maintained by serial passages *in vivo* at 3 week intervals. We chose the intramuscular implant because it produces a more homogeneous tumour take and tumour growth than the subcutaneous implant. In fact, 7–8 days after cell injection, a tumour mass was evident in 100% of injected mice. Single-cell suspensions were prepared from tumours by enzymatic digestion as previously described (Arancia *et al.*, 1991). Each experimental group consisted of 10–12 animals. About 70 animals were used for a single experiment. CDDP treatment was started at day 4 after tumour implant, by intraperitoneal (i.p.) administration of 10% of the lethal dose ($LD_{10}$), calculated according to the Spearman and Karber method (Finney, 1964), subdivided into three daily injections of 3.3 mg kg $day^{-1}$ (days 4–6). (S)ODNs were administered intravenously (i.v.) at a dose of 1 mg/mouse $day^{-1}$ (days 7–14). The following schedules were employed: (a) antisense (S)ODNs × 8 days; (b) sense (S)ODNs × 8 days; (c) CDDP × 3 days; (d) CDDP × 3 days followed by antisense (S)ODNs × 8 days; (e) CDDP × 3 days followed by sense (S)ODNs × 8 days. Mice in the control group received 0.2 ml of 0.9% sodium chloride solution i.p. × 3 days and i.v. × 8 days. Tumour growth was monitored daily by caliper measurements of length and width. Tumour weight was calculated as follows (Geran *et al.*, 1972):

$$\text{tumor weight (mg)} = [\text{length (mm)} \times \text{width (mm)}^2]/2$$

The effect of treatment on tumour growth was assessed using the following end points: (1) tumour weight inhibition (TWI%); (2) tumour growth delay (T-C, days); (3) median time (days) of appearance of palpable tumours in treated and control group.

*Statistical analysis*

Statistical differences from values of controls and of other groups were assessed by the Mann–Whitney non-parametric method.

### Results

*c-*myb *mRNA and protein levels in c-*myb *(S)ODNs-treated LoVo Dx cells*

We evaluated c-*myb* mRNA expression in LoVo Dx cells immediately after treatment with c-*myb* sense or antisense (S)ODNs (Figure 1a). In cells treated with c-*myb* antisense (S)ODNs there was a marked reduction in c-*myb* mRNA levels (about 80%, by densitometric analysis and normalisation to β-actin mRNA). No decrease in c-*myb* mRNA levels was detected after treatment with c-*myb* sense (S)ODNs. Western blot analysis was then performed to assess Myb protein levels in (S)ODN-treated cells. Eight hours after the beginning of (S)ODN treatment, there was no difference in Myb protein levels between control, sense- or antisense-treated cells (data not shown). A decrease in Myb protein level was detected after 30 h of exposure to antisense (S)ODNs (39% decrease relative to controls, by densitometry analysis). A greater reduction in Myb protein levels (65% decrease relative to controls, by densitometry analysis) was evident after 72 h treatment (Figure 1b).

*Effect of c-*myb *(S)ODNs on LoVo Dx cell proliferation*

The ability of c-*myb* (S)ODNs to inhibit proliferation of LoVo Dx cells was assessed by direct cell countings, and by cell cycle analysis. Cell counts of cultures treated with 18-mer c-*myb* antisense (S)ODNs indicated a reduction in cell proliferation at day 3, and a more pronounced inhibition (50–60% of reduction) from the days 6–10 of culture (Figure 2a). Treatment with sense (S)ODNs did not affect proliferation of LoVo Dx cells (Figure 2a). The 18-mer c-*myb* (S)ODNs had no significant effect on LoVo Dx cell survival,

**Figure 1** Expression of c-*myb* mRNA (**a**) and protein (**b**) in LoVo Dx cells treated with c-*myb* (S)ODNs. LoVo Dx cells were untreated (C) or exposed for 3 consecutive days to 10 μM c-*myb* sense (S) or antisense (A) oligodeoxynucleotides starting 24 h after seeding the cells in culture. To evaluate the expression of c-*myb* mRNA, cells were harvested at the end of treatment, and total RNA was isolated and divided into two equal portions that were separately amplified by RT–PCR with c-*myb* and β-actin-specific primers. Western blotting analysis was performed 30, and 72 h after the beginning of treatment with (S)ODNs. Representative of three independent experiments with similar results.

in the same experimental conditions. A clonogenic assay performed at each day of growth, starting from days 2–10, demonstrated that cell survival was not affected in the case of c-*myb* sense treatment whereas after c-*myb* antisense treatment the decrease in cell survival was 25% (on day 2), 30% (on day 5) and 30% (on day 10) (data not shown). Additional experiments were performed with a 15-mer c-*myb* antisense ODN and with two three-base-mismatched derivatives, one of which included (see Materials and methods) the 4-G motif reported to non-specifically inhibit proliferation of certain cell lines (Yaswen *et al.*, 1993; Burgess *et al.*, 1995). The c-*myb* antisense (S)ODNs inhibited LoVo Dx cell proliferation, whereas neither mismatched sequence had appreciable effects (Figure 2b).

Cell cycle phases distribution of LoVo Dx cells exposed to sense or antisense c-*myb* (S)ODNs was evaluated by flow cytometry (Figure 3). The analysis was performed on days 1, 3, 6 and 9 of growth in liquid culture. Treatment with c-*myb* antisense (S)ODNs resulted in accumulation of cells in the $G_1$ phase of the cell cycle with a simultaneous reduction of cells in the S-phase, already detectable on the third day of culture. No clear effect on the $G_2+M$ compartment was observed. Cell cycle distribution of LoVo Dx cells treated with c-*myb* sense (S)ODNs was indistinguishable from that of untreated cells (data not shown). Together, these results indicate that treatment with c-*myb* antisense (S)ODNs induces inhibition of cell proliferation, evident only several days after exposure to the (S)ODNs.

*Effect of c-*myb *antisense (S)ODNs and DDP treatment on LoVo Dx cell proliferation and c-*myb *mRNA levels*

Figure 4a shows the effect of CDDP and c-*myb* antisense (S)ODNs given alone or in combination on LoVo Dx cell proliferation. Incubation for 2 h with CDDP followed by a 3 day c-*myb* antisense (S)ODN treatment led to an increase in sensitivity of LoVo Dx cells to CDDP. In fact, as evaluated after 6–8 days of culture, the (S)ODN/CDDP combination produced an approximately 80% inhibition of cell proliferation whereas CDDP and (S)ODNs, used individually, produced approximately a 50% and 55% inhibition respectively. The antiproliferative effect of the various compounds was correlated with levels of c-*myb* mRNA, detected by RT–PCR. Only the treatment with c-*myb* antisense (S)ODNs, individually or in combination with CDDP, induced a decrement of c-*myb* mRNA levels (Figure 4b).

*Isobologram analysis*

To define the type of the interaction and the interaction index (Berembaum, 1981, 1985) between CDDP and c-*myb* antisense (S)ODNs in combination we determined, initially, the effect of increasing doses of CDDP (Figure 5a) and c-*myb* antisense (S)ODNs (Figure 5b) given separately, in terms of mean cell number at the 5, 8 and 10 days of growth. Then, we evaluated the effect, in terms of mean cell number at the same days of growth, of the two agents given in combination (Figure 5c) according to the schedules reported in the Materials and methods section. For each value in Figure 6c, the interaction index I (Berembaum, 1981) was calculated as follows:

$$I = A_C/A_E + B_C/B_E$$

where $A_C$ and $B_C$ are the dosages of the agents used in combination eliciting a certain effect while $A_E$ and $B_E$ are respectively the dosages of the agents A and B eliciting the same effect when administered alone.

Single doses of CDDP and c-*myb* antisense (S)ODNs producing the same effect on cell growth at the same day, in terms of mean cell number, were determined, interpolating the results from Figure 5a and b. When the dose of the agent used alone and producing the same effect of the combination exceeds the highest experimental dose, this maximum dose value is used to calculate the interaction index. Table I shows the interaction index for the combination CDDP/c-*myb* antisense calculated as above from the data reported in Figure 5c. According to Berembaum (1981), the evaluation of the type and level of interaction among agents can be approached in terms of interaction index (I), defined as the sum of the ratios between dosages of the agent which, when used in combination or alone, induces the same effect in terms of cell survival or growth. The interaction index can assume the following values:

$I < 1$ indicates synergistic interaction;

$I = 1$ indicates additive interaction;

$I > 1$ indicates antagonistic interaction.

As the interaction index values reported in Table I ranging around 1 and taking into account the uncertainty related to the experimental data (Gentile *et al.*, 1992), the interaction index value for additivity can be included in the range 0.8–1.2. Therefore, from our experimental data we can conclude

**Figure 2** Effect of c-*myb* (S)ODNs on LoVo Dx cells proliferation. (**a**) Treatment with 18-mer c-*myb* sense (▲) or antisense (■) (S)ODNs (10 μM) was performed for 3 consecutive days starting 24 h after seeding the cells in culture. Control cells (●) were left untreated. Cell counts and viability were determined daily until day 10 of culture. Representative of three different experiments with similar results. (**b**) Treatment with a 15-mer c-*myb* antisense ODN (■) or two three-base-mismatched (S)ODNs (▲, ○), and cell counts of untreated (●) and (S)ODN-treated cells were as described above. Representative of three different experiments with similar results. Both in (**a**) and (**b**), each value is an average ± standard error (s.e.) of four different determinations within the same experiment. When not shown, the standard error is smaller than the symbol.

**Figure 3** Cell cycle distribution of LoVo Dx cells treated with c-*myb* antisense (S)ODNs. Treatment with c-*myb* (S)ODNs (10 μM) was carried out for 3 consecutive days starting 24 h after seeding the cells in culture. DNA content analysis was performed on days 1, 3, 6 and 9 of culture. At least duplicate samples were analysed for each day; 20 000 events were scored for each sample. The effect of c-*myb* sense (S)ODNs on cell cycle phase distribution was not reported as it was similar to that observed for control, untreated cells. -■-, antisense $G_1$ phase; -□-, contol $G_1$ phase; -▲-, antisense S-phase; -△-, control S-phase; -●-, antisense $G_2$+M phase; -○-, control $G_2$+M phase. Results represent the mean ± standard error obtained from three independent experiments with different (S)ODN preparations.

that the combination between CDDP and c-*myb* antisense produces an additive interaction.

### In vivo *studies*

The anti-tumour activity of c-*myb* (S)ODNs and CDDP, alone or in combination, was evaluated in nude mice bearing LoVo Dx solid tumours (Table II). Administration of c-*myb* sense (S)ODNs was completely ineffective against LoVo Dx, the mean weight of the tumour in the treated group being the same as that of the control group. Treatment with antisense (S)ODNs, on the other hand, inhibited local tumour growth, as compared with the untreated group. In fact, at the end of treatment (14 days post implant) tumour weights of control and antisense groups were 197±27 mg (mean±s.d.) and 120±35 mg respectively ($P<0.01$). No toxicity was noted; the modest loss in body weight (5% reduction) noted during the treatment, was made up after treatment. The response of

**Figure 4** Effect of c-*myb* antisense (S)ODNs and CDDP on LoVo Dx cell proliferation and c-*myb* mRNA levels. (**a**) c-*myb* antisense (S)ODNs were added to the culture medium at 10 μM day$^{-1}$ for 3 consecutive days. CDDP treatment (2 μg ml$^{-1}$ for 2 h) was started 24 h after seeding the cells in culture. For antisense–drug combinations, c-*myb* antisense (S)ODNs were added to the culture medium at 10 μM day$^{-1}$ for 3 consecutive days after CDDP treatment (2 μg ml$^{-1}$ for 2 h). Control cells were cultured in the same conditions without (S)ODNs. Treatment with c-*myb* sense (S)ODNs was without effects and treatment with c-*myb* sense/CDDP was similar to CDDP alone (not shown). -●-, Control; -■-, antisense; -▲-, cisplatin; -□-, cisplatin→antisense. Representative of three independent experiments with similar results. Each value is an average±s.e. of four different determinations within the same experiment. When not shown, the standard error is smaller than the symbol. (**b**) To assess c-*myb* mRNA levels, cells were harvested at the indicated times from the beginning of treatment, and total RNA was isolated and divided into two equal portions (0.5 μg each) separately amplified by RT–PCR (30 cycles) with c-*myb* and β-acTin-specific primers. Following hybridisation with $^{32}$P-labelled specific probes, c-*myb* and β-actin blots were exposed to X-ray films for 4 and 2 h respectively. Lanes are as follows: (1) untreated cells; (2) c-*myb* sense (S)ODN-treated cells; (3) c-*myb* antisense (S)ODN-treated cells; (4) CDDP-treated cells; (5) CDDP-c-*myb* sense (S)ODN-treated cells; (6) CDDP-c-*myb* antisense (S)ODN-treated cells. Results are from a representative experiment.

**Figure 5** Dose-dependent effect of CDDP, c-*myb* antisense (S)ODNs and CDDP/c-*myb* antisense (S)ODNs combination on LoVo Dx cell growth. In (**a**), (**b**) and (**c**) cells were treated 24 h after seeding according to the following schedules. (**a**) CDDP for 2 h: a, 1 μg ml$^{-1}$; b, 2 μg ml$^{-1}$; c, 4 μg ml$^{-1}$; d, 6 μg ml$^{-1}$. (**b**) c-*myb* antisense for 3 consecutive days: a, 2.5 μM day$^{-1}$; b, 5 μM day$^{-1}$; c, 10 μM day$^{-1}$; d, 20 μM day$^{-1}$. (**c**) CDDP for 2 h followed by c-*myb* antisense for 3 consecutive days: a, CDDP 1 μg ml$^{-1}$ + c-*myb* antisense 5 μM day$^{-1}$; b, CDDP 1 μg ml$^{-1}$ + c-*myb* antisense 10 μM day$^{-1}$; c, CDDP 2 μg ml$^{-1}$ + c-*myb* antisense 5 μM day$^{-1}$; d CDDP 2 μg ml$^{-1}$ + c-*myb* antisense 10 μM day$^{-1}$. Cell counts were determined as described in Material and methods at days 5 (□), 8 (▨) and 10 (■) of cell culture. Representative of two separate experiments with similar results. Each value is an average±s.e. of four different determinations within the same experiment.

**Table I** Interaction Index of the combination CDDP + c-*myb* antisense (S)ODNs

| *Combination* | *Growth days* 5 | 8 | 10 |
|---|---|---|---|
| CDDP 1 μg ml$^{-1}$ + AS 5 μM | 0.98 | 0.99 | 1.18 |
| CDDP 1 μg ml$^{-1}$ + AS 10 μM | 0.96 | 0.84 | 0.98 |
| CDDP 2 μg ml$^{-1}$ + AS 5 μM | 1.26 | 1.03 | 0.98 |
| CDDP 2 μg ml$^{-1}$ + AS 10 μM | 1.08 | 1.01 | 0.98 |

**Table II** Anti-tumour effects of c-*myb* antisense (S)ODNs and CDDP on LoVo Dx implanted in nude mice

| *Schedule*[a] | *TWI*[b] (%±s.d.) | *T-C*[c] (days±s.d.) | *Body weight*[d] *loss (%)* | *Drug deaths*[e] |
|---|---|---|---|---|
| Antisense | 39±10 | 3.5±0.5 | 5 | 0/20 |
| Sense | 8±2 | 0.5±0.1 | 4 | 0/20 |
| CDDP | 41±12 | 5±1.1 | 13 | 2/24 |
| CDDP plus antisense | 62±21 | 9±2 | 12 | 2/24 |
| CDDP plus sense | 35±6 | 5±1 | 15 | 1/24 |

[a] Mice received: c-*myb* sense or antisense (S)ODNs (1 mg/mouse - day$^{-1}$ i.v.) on days 7–14 after tumour implant; CDDP i.p. (3.3 mg kg day$^{-1}$, i.p.) on days 4–6; CDDP (3.3 mg kg day$^{-1}$, i.p.) on days 4–6 followed by c-*myb* sense or antisense (S)ODNs (1 mg/mouse day$^{-1}$, i.v.) on days 7–14 after tumour implant. [b] Tumour weight inhibition: mean tumour weight of the treated groups divided by mean tumour weight of the control group, minus 1 × 100. [c] Tumour growth delay: median time in days for the treated (T) groups to reach 250 mg minus median time in days for the control (C) group to reach the same size. [d] Maximum body weight loss during the experimental period. [e] Deaths ocurring in treated mice 1 week after the end of treatment were considered as due to drug toxicity. Results represent the mean of two independent experiments.

LoVo Dx tumour to CDDP treatment was similar to that observed after antisense (S)ODN injection, the mean tumour weight being $118 \pm 22$ mg. The CDDP/(S)ODNs combination determined a marked local control of tumour growth, with a TWI of 62% and a growth delay of 9 days being obtained. In particular, tumour weights measured after treatment with CDDP and antisense (S)ODNs were significantly smaller ($80 \pm 23$ mg) than those from the untreated, CDDP, or antisense (S)ODN-treated groups. Statistical analysis showed a significant difference in tumour weight in mice receiving no treatment or treated with CDDP and/or (S)ODNs. In fact, a $P<0.01$ was observed comparing the tumour weight of CDDP/(S)ODN-treated mice *vs* that in the untreated group; a $P<0.05$ was obtained comparing the tumour weight of CDDP/(S)ODN- and (S)ODN- or CDDP-treated groups. The toxicity observed in combination experiments, both in terms of animal death and body weight loss, was superimposable to that observed after CDDP treatment.

### Discussion

The purpose of this study was to evaluate the efficacy of the CDDP/c-*myb* antisense (S)ODN combination on the *in vitro* and *in vivo* growth of LoVo Dx, a human colon carcinoma cell line with high levels of c-*myb* expression (Melani *et al.*, 1991). It has been postulated that the c-*myb* gene is involved in the pathogenesis of colon carcinoma (Alitalo *et al.*, 1984; Torelli *et al.*, 1987; Ramsay *et al.*, 1991), suggesting that the ability to selectively down-regulate this expression, by use of antisense ODNs, might have therapeutical potential. An obstacle to a widespread use of unmodified antisense ODNs is represented by their short half-life in biological systems (Stein *et al.*, 1988). Accordingly, for both *in vitro* and *in vivo* experiments, we used phosphorothioate oligodeoxynucleotides, which are more resistant to cleavage by nucleases (Stein *et al.*, 1988; Agrawal *et al.*, 1991). Moreover, (S)ODNs are characterised by a good solubility in aqueous solution and an efficient hybridisation with mRNA, making them possible therapeutic agents for eventual clinical application.

We selected c-*myb* antisense (S)ODNs corresponding either to codons 2–7 or 2–6 as the region immediately downstream from the initiation codon is commonly targeted in antisense experiments and as we were previously able to inhibit proliferation of a human leukaemia cell line by targeting the same mRNA region (Citro *et al.*, 1992). Here, we demonstrated that c-*myb* expression plays an important role in LoVo Dx cell proliferation, both *in vitro* and *in vivo*. We observed that a down-regulation of c-Myb protein is evident as early as 30 h after the beginning of treatment when cell proliferation was only marginally affected. The early down regulation of c-Myb protein levels after antisense (S)ODN treatment most likely reflects the short half-life (30–60 min) of c-Myb mRNA and protein (Thiele *et al.*, 1988; Lipsick and Boyle, 1987), and is consistent with the interpretation that the down-regulation of c-myb protein expression is not the result but the cause of the reduction in cell proliferation.

We also found that *in vivo* treatment of LoVo Dx tumour with c-*myb* antisense (S)ODNs reduces the tumour growth, in terms of both tumour weight inhibition and tumour growth delay. These data agree with those of other authors, confirming the *in vivo* antiproliferative effect of c-*myb* antisense (S)ODNs and its potential usefulness for the systemic treatment of solid and non-solid tumours (Ratajczak *et al.*, 1992; Hijiya *et al.*, 1994). In particular, our results are similar to those obtained in the experimental melanoma model (Hijiya *et al.*, 1994); whereas a higher response was reported using a mouse model of human leukaemia (Ratajczak *et al.*, 1992), probably reflecting cell type differences or the higher sensitivity of haematopoietic cells to c-*myb* deprivation.

As demonstrated by the isobologram analysis, the combination of c-*myb* antisense (S)ODNs with CDDP produces an additive effect on LoVo Dx cell proliferation *in vitro*, if compared with the effect produced by the two agents given alone. In addition, these *in vitro* results are supported by the *in vivo* data, where a significant difference between the tumour weight inhibition in mice treated with the combination CDDP/c-*myb* antisense and the tumour weight inhibition in mice treated with the two agents given separately was observed.

Most probably, CDDP exerted its antiproliferative activity independently of a direct effect on c-*myb* expression, as c-*myb* mRNA levels were not down-regulated in cultures treated with CDDP alone (Figure 4b). Incidentally, this finding is an additional proof that the down-regulation of c-*myb* expression is a specific consequence of the treatment with c-*myb* antisense (S)ODNs, and not of the inhibition of cell proliferation *per se*. It has already been demonstrated that the degree of resistance to CDDP correlates directly with the level of c-*myc* expression (Sklar and Prochownik, 1991) and that the treatment with c-*myc* antisense ODNs leads to an increased CDDP sensitivity in a small-cell lung carcinoma line that shows c-*myc* mRNA and protein overexpression, this effect not being evident in CDDP-sensitive cells (Prins *et al.*, 1993). It has also been demonstrated that the expression of a *fos* ribozyme, which reduces Fos protein synthesis, enhances the sensitivity of a human ovarian cancer line resistant to CDDP to antineoplastic agents, including cisplatin (Funato, 1994). Moreover, the combination of conventional chemotherapeutic agents and antisense against *bcr/abl* or c-*myb* or the combination of antitumour cytotoxic T lymphocytes and c-*myb* antisense oligodeoxynucleotides has been reported to be highly effective in killing tumour cells (Nieborowska-Skorska *et al.*, 1994). Thus, the level of expression of genes involved in cell proliferation (e.g. c-*myc*, c-*myb*, c-*fos*) might affect the therapeutic response of malignant cells.

In conclusion, our data support the potential use of (S)ODNs in combination with antineoplastic agents to improve cancer management.

Furthermore, it is noteworthy that, even if the two drugs are only additive in their lethal effects, as the toxic effects are less than additive a net gain in the therapeutic index may be achieved that is clinically exploitable (Drewinko *et al.*, 1976).

### Abbreviations

CDDP, cisplatin; (S)ODNs, phosphorothioate oligodeoxynucleotides; RT–PCR, reverse transcriptase–polymerase chain reaction; TWI, tumour weight inhibition; T-C, tumour growth delay.

### Acknowledgements

We thank Anna Biroccio and Carmela Amedeo for their expert technical help and Gail Ayers for editorial assistance. CC was supported in part by an AIRC (Italian Association for Cancer Research) fellowship. This work was supported by grants from the Italian Ministry of Public Health, the Italian Association for Cancer Research, the National Research Council, ACRO no. 93.02348 F.39, and the American Cancer Society.

### References

AGRAWAL S, TEMSAMANI J AND TANG YJ. (1991). Pharmacokinetics, biodistribution and stability of oligodeoxynucleotide phosphorothioate in mice. *Proc. Natl Acad. Sci. USA*, **88**, 7595–7599.

ALITALO K, WINQVIST R, LIN CC, DE LA CHAPELLE A, SCHWAB M AND BISHOP JM. (1984). Aberrant expression of an amplified c-*myb* oncogene in two cell lines from a colon carcinoma. *Proc. Natl Acad. Sci. USA*, **81**, 4534–4538.

ARANCIA G, LEONETTI C, MALORNI W, GRECO C, FORMISANO G, MARANGOLO M AND ZUPI G. (1991). Different effects of sequential combinations of N-methylformamide with 5-fluouracil on human colon carcinoma cells growing in nude mice. *J. Cancer Res. Clin. Oncol.*, **117**, 351–358.

BERENBAUM MC. (1981). Criteria for analysing interaction between biologically active agents. *Adv. Cancer Res.*, **35**, 269–335.

BERENBAUM MC. (1985). The expected effects of a combination of agents: the general solution. *J. Theor. Biol.*, **114**, 413–432.

BURGESS TL, FISCHER EF, ROSS SL, BREADY JV, QIANY-X, BAYEWITCH LA, COHEN AM, HENERE CJ, HU SS-F, KRAMER TB, LOTT FD, MARTIN FH, PIERCE GF, SIMONET L AND FARRELL CL. (1995). The antiproliferative activity of c-myb and c-myc antisense oligonucleotides in smooth muscle cells is caused by a nonantisense mechanism. *Proc. Natl Acad. Sci. USA*, **92**, 4051–4055.

CALABRETTA B. (1991). Inhibition of protooncogene expression by antisense oligodeoxynucleotides: biological and therapeutic implications. *Cancer Res.*, **51**, 4505–4510.

CALABRETTA B AND NICOLAIDES NC. (1992). c-myb and growth control. Critical reviews in eukaryotic gene expression. *Gene Expression*, **2**, 225–235.

CHOMCZYNSKI P AND SACCHI N. (1987). Single-step method of RNA isolation by acid guandidium thiocyanate-phenol-chloroform extraction. *Anal. Biochem.*, **162**, 156–159.

CITRO G, PERROTTI D, CUCCO C, D'AGNANO I, SACCHI A, ZUPI G AND CALABRETTA B. (1992). Inhibition of leukemia cell proliferation by receptor-mediated uptake of c-myb antisense oligodeoxynucleotides. *Proc. Natl Acad. Sci. USA*, **89**, 7031–7035.

CITRO G, SZCZYLIK C, GINOBBI P, ZUPI G AND CALABRETTA B. (1994). Inhibition of leukemia cell proliferation by folic acid-polylysine-mediated introduciton of *c-myb* antisense oligodeoxynucleotides into HL60 cells. *Br. J. Cancer*, **69**, 463–467.

DREWINKO B, LOO TL, BROWN B, GOTTLIEB JA AND FREIREICH EJ. (1976). Combination chemotherapy *in vitro* with adriamycin. Observation of additive, antagonistic, and synergistic effects when used in two-drug combination on cultured human lymphoma cells. *Cancer Biochem. Biophys.*, **1**, 187–195.

FEARON ER AND VOGELSTEIN BA. (1990). A genetic model for colorectal tumorigenesis. *Cell*, **61**, 759–767.

FINNEY DJ. (1964). *Statistical Methods in Biological Assay*, 2nd edn, pp. 525–529. Charles Griffin: London.

FOX MH. (1980). A model for the computer analysis of synchronous DNA distributions obtained by flow cytometry. *Cytometry*, **1**, 71–75.

FUNATO T. (1994). Circumventing drug resistance in human tumors by antisense rybozyme. *Gan To. Kagaku Ryoho*, **21**, 336–342.

GENTILE FP, CHIATTI L, MAURO F, BRIGANTI G, FLORIDI A AND BENASSI M. (1992). Interaction of cytotoxic agents: a rule - based system for computer–assisted cell survival analysis. *Anticancer Res.*, **12**, 637–644.

GERAN RI, GREENBERG NH, MACDONALD MM, SHUMACHER AM AND ABBOT BJ. (1972). Protocols for screening chemical agents and natural products against animals tumours and other biological systems (3rd edn). *Cancer Chemother. Rep.*, **3**, 1–88

GRANDI M, GERONI C AND GIULIANI FC. (1986). Isolation and characterization of a human colon adenocarcinoma cell line resistant to doxorubicin. *Br. J. Cancer*, **54**, 515–518.

HIJIYA N, ZHANG J, RATAJCZAK MZ, KANT JA, DERIEL K, HERLYN M, ZON G AND GEWIRTZ AM. (1994). Biologic and therapeutic significance of MYB expression in human melanoma. *Proc. Natl Acad. Sci. USA*, **91**, 4499–4503.

LIPSICK JS AND BOYLE WJ. (1987). c-myb protein expression is a late event during T-lymphocyte activation. *Mol. Cell. Biol.*, **7**, 3358–3360.

MAJELLO B, KENYON LC AND DALLA FAVERA R. (1986). Human c-*myb* proto-oncogene: nucleotide sequence of c-DNA and organization of the genomic locus. *Proc. Natl Acad. Sci. USA*, **83**, 9636–9640.

MELANI C, RIVOLTINI L, PARMIANI G, CALABRETTA B AND COLOMBO MP. (1991). Inhibition of proliferation by c-*myb* antisense oligodeoxynucleotides in colon adenocarcinoma cell lines that express c-*myb*. *Cancer Res.*, **51**, 2897–2901.

NEGRONI A, SCARPA S, ROMEO A, FERRARI S, MODESTI A AND RASCHELLÀ G.(1991). Decrease of proliferation rate and induction of differentiation by a MYCN antisense DNA oligomer in a human neuroblastoma cell line. *Cell Growth Diff.*, **2**, 511–518.

NIEBOROWSKA-SKORSKA M, NAKASHIMA M, RATAJCZAK M, STEPLEWSKI Z, CALABRETTA B AND SKORSKI T. (1994). Oncogene-targeted antisense oligodeoxynucleotides combined with chemotherapy or immunotherapy: a new approach for tumor treatment? *Folia Histochem. Cytobiol.*, **32**, 35–40.

PRINS J, DE VRIES EGE, PROFFIT RT AND MULDER NH. (1993). The effect of antisense c-myc oligonucleotides on proliferation and cisplatin (CDDP) sensitivity of a small cell lung carcinoma cell line (GLC4) and its 5-6x cDDP-resistant subline (GLC4-cDDP). *Proc. Am. Ass. Cancer Res.*, **34**, 279–283.

RAMSAY RG, THOMPSON MA, HAYMAND JA, REID G, GONDA TJ AND WHITEHEAD RH. (1992). Myb expression is higher in malignant human melanoma colonic carcinoma and premalignant adenomatous polyps than in normal mucosa. *Cell Growth Diff.*, **3**, 723–730.

RASCHELLÀ G, NEGRONI A, SKORKI T, PUCCI S, NIEBOROWSKA-SKORSKA M, ROMEO A AND CALABRETTA B. (1992). Inhibition of proliferation by c-*myb* antisense RNA and oligodeoxynucleotides in transformed neuroectodermal cell lines. *Cancer Res.*, **52**, 4221–4226.

RATAJCZAK MZ, KANT JA, LUGER S-M, HIJIYA N, ZHANG J, ZON G AND GEWIRTZ AM. (1992). *In vivo* treatment of human leukemia in a scid mouse model with c-*myb* antisense oligodeoxynucleotides. *Proc. Natl Acad. Sci. USA*, **89**, 11823–11827.

ROSENBERG B. (1985). Fundamental studies with cisplatin. *Cancer*, **55**, 2303–2316.

ROWINSKY EK, GILBERT MR, MCGUIRE WP, NOE DA, GROCHOW LB, FORASTIERE AA, ETTINGER DS, LUBEJKO BG, CLARK B, SARTORIUS SE, CORNBLATH DR, HENDRICKS CB AND DONEHOWER RC. (1991). Sequences of taxol and cisplatin: a phase I and pharmacologic study. *J. Clin. Oncol.*, **9**, 1692–1703.

SCHEITHAUER W, ROSEN H, SCHIESSEL R, SCHULLER J, KARALL M, ERNST F, SEBESTA C, KORNEK G, HENTSCHEL E, MARCZELL A AND DEPISCH D. (1991). Treatment of patients with advanced colorectal cancer with cisplatin, 5-fluorouracil, and leucovorin. *Cancer*, **67**, 1294–1298.

SHEN-ONG GLC. (1990). The c-*myb* oncogene. *Biochim. Biophys. Acta*, **32**, 49–52.

SKLAR MD AND PROCHOWNIK EV. (1991). Modulation of cisplatinum resistance in Friend erytroleukemia cells by c-myc. *Cancer Res.*, **51**, 2118–2123.

STEIN CA, SUBASINGHE C, SHINOZUKA K AND COHEN JS. (1988). Physicochemical properties of phosphorothioate oligodeoxynucleotides. *Nucleic Acids Res.*, **16**, 3209–3221.

THIELE CJ, COHEN PS AND ISRAEL M. (1988). Regulation of c-myb expression in human neuroblastoma cells during retinoic acid-induced differentiation. *Mol. Cell. Biol.*, **8**, 1677–1683.

TORELLI G, VENTURELLI D, COLÒ A, ZANNI C, SELLERI L, MORETTI L, CALABRETTA B AND TORELLI U. (1987). Expression of c-*myb* proto-oncogene and other cell cycle related genes in normal and neoplastic colonic mucous. *Cancer Res.*, **47**, 5266–5269.

VENTURELLI D, TRAVALI S AND CALABRETTA B. (1990). Inhibition of T-cell proliferation by a c-myb antisense oligomer is accompanied by selective down-regulation of DNA polymerase a expression. *Proc. Natl Acad. Sci. USA*, **87**, 5963–5967.

YASWEN P, STAMPFER MR, GHOSH K AND COHEN JS. (1993). Effects of sequence of thioated oligonucleotides on cultured human mammary epithelial cells. *Antisense Res. and Dev.*, **3**, 67–77.

YOKOYAMA K AND IMAMOTO F. (1987). Transcriptional control of the endogenous MYC protooncogene by antisense RNA. *Proc. Natl Acad. Sci. USA*, **84**, 7363–7367.